# Detachment of Dodecane from Silica Surfaces with Variable Surface Chemistry Studied Using Molecular Dynamics Simulation

**DOI:** 10.3390/molecules28124765

**Published:** 2023-06-14

**Authors:** Binbin Jiang, Huan Hou, Qian Liu, Hongyuan Wang, Yang Li, Boyu Yang, Chen Su, Min Wu

**Affiliations:** 1 State Key Laboratory of Water Resource Protection and Utilization in Coal Mining, China Energy Investment Group, Beijing 102211, China; 20029907@chnenergy.com.cn (B.J.);; 2 School of Chemical and Environmental Engineering, China University of Mining and Technology (Beijing), Beijing 100083, China

**Keywords:** n-dodecane, SiO_2_ surface, adsorption, detachment, MD simulation

## Abstract

The adsorption and detachment processes of n-dodecane (C_12_H_26_) molecules were studied on silica surfaces with variable surface chemistry (Q^2^, Q^3^, Q^4^ environments), using molecular dynamics simulations. The area density of the silanol groups varied from 9.4 to 0 per nm^2^. The shrinking of the oil–water–solid contact line was a key step for the oil detachment, due to water diffusion on the three-phase contact line. The simulation results showed that oil detachment was easier and faster on a perfect Q^3^ silica surface which had (≡Si(OH))-type silanol groups, due to the H-bond formation between the water and silanol groups. When the surfaces contained more Q^2^ crystalline type which had (≡Si(OH)_2_)-type silanol groups, less oil detached, due to the formations of H-bonds among the silanol groups. There were no silanol groups on the Si-OH 0 surface. Water cannot diffuse on the water–oil–silica contact line, and oil cannot detach from the Q^4^ surface. The detachment efficiency of oil from the silica surface not only depended on the area density, but also on the types of silanol groups. The density and type of silanol groups depend on the crystal cleavage plane, particle size, roughness, and humidity.

## 1. Introduction

The displacement mechanism of oil from solid surfaces plays a crucial role in a variety of technological applications such as enhanced oil recovery, self-cleaning materials, and flotation. Although the operating parameters and methods are different during the technological processes, two common essential and fundamental steps are involved, which are the liberation of oil from reservoir solids or host rocks, and the separation of oil and water [1,2]. The interface properties of the solid–oil–water system determine the oil detachment from the rock surfaces [3,4,5,6,7].

Due to the different intrinsic composition and structure of reservoir rocks, the reservoir rocks exhibit different characteristic properties and wettability. In general, the reservoir rocks can be divided into the hydrophobic type (carbonate rocks) and the hydrophilic type (silicate rocks). Experimental studies have mostly focused on hydrophilic mineral surfaces, specifically adsorption and wettability studies for crude oil extraction, mineral flotation, removal of contaminants, etc. [8,9,10]. The hydrophobicity of mineral surfaces is an important factor which affects the adsorption and detachment of crude oil, and the altering of the solid wettability can affect the oil recovery rate.

Nowadays, molecular dynamics (MD) simulations have been used to evaluate complex interactions from an atomistic point of view, and provide comprehensive information about the static and dynamic properties of the system at the molecular level [11]. For example, MD simulations were used to study the aggregation mechanism of asphaltenes on the oil–water interface and in different types of solvents, the effect of different surfactant structure, the force field on the interfacial properties of water and oil, and the wettability variation of reservoir rocks at the molecular scale [12].

Liu et al. [13] firstly evaluated intermolecular interactions in oil–water surfactant hydrophilic silica systems, using MD simulations. Their simulation results demonstrated the mechanism of oil detachment from silica surfaces in the presence of dodecyl trimethyl ammonium bromide (DTAB). A three-stage model was revealed, which was composed of the formation of water channels, the diffusion of water on the solid surface, and the solubilization of alkane molecules in surfactant micelles. Surfactant molecules can lower interfacial tension between the oil and water interface, accompany water molecules via their tail or head groups into a water channel, and facilitate the process of oil detachment from a rock surface. Due to the H-bonding and electrostatic interaction, water diffused on the oil interface, and formed a gel layer on the hydrophilic silica surface. Oil molecules were completely removed and solubilized into the surfactant micelles.

The adsorption of surfactant molecules altered the wetting properties of the solid surface, which was important for an enhanced oil recovery. Moncayo-Riascos et al. [14,15,16] investigated how the ester groups of surfactants could interact with the rock surface and change its wettability. The contact angles of water were measured on the amorphous silica coated by six different organosilicon surfactants with different chain lengths. In addition, they evaluated the process of wettability alteration on glass surfaces in the presence of surfactants. The adsorption of surfactants onto the glass surfaces could change the surface wettability. Zhang et al. [17] evaluated the effect of ethoxylate group numbers of a non-anionic surfactant on the wettability alteration of a lignite surface. Based on their simulation results, it was found that when the surfactants had more ethoxylate groups, lignite surfaces became more hydrophobic after the adsorption of non-anionic surfactants.

In contact with crude oil, the rock surface changed into a hydrophobic one, due to the adsorption of polar components. Liu et al. [18] investigated whether the {1 0 − 1 4} surface of calcite in carbonate reservoirs was indeed water-wet under initial conditions. The contact angle of the calcite surface was 68.47 ± 3.6 deg. However, when the reservoirs came into contact with the crude oil, the components containing multiple hydroxyl functional groups, such as glycerol (GLYC), were adsorbed onto the water-wet calcite surface by hydrogen bonding and Coulomb interaction, and the alcite surfaces became hydrophobic. Mohammadail et al. [19] used MD simulation to evaluate the wettability alteration under a steam injection process for bitumen and heavy oil recovery. The simulation results showed that when there were more asphaltenes, the adsorption energy was higher between the bitumen/heavy oil and quartz surfaces, due to the Coulomb interactions. Additionally, quartz surfaces became more oil-wet when temperatures were above the water boiling temperature. They were extremely water-wet at ambient conditions.

The wettability alteration process on different types of reservoir rocks was studied by Mohammed and Gadikota through MD simulations [20]. Three rock surfaces, including illite, calcite and quartz, were constructed to evaluate the process of wettability alteration in contact with asphaltene molecules. The illite was the most hydrophobic among these three surfaces. Time-dependent self-diffusion of asphaltene monomers was realized, and played a crucial role in the wettability alteration process. Li et al. [21] studied the oil detachment process from four different types of rock surfaces, including quartz, siderite, calcite and dolomite. The wettability of dolomite rock surfaces was changed by all types of surfactants. However, the non-anionic one took the longest time among the other surfactants. The water channel formed quicker in the case of the anionic surfactant than with other types of surfactants.

The wetting characteristics of water were investigated on three typical inorganic minerals (calcite, quartz and montmorillonite) by Yang et al. [22]. The water–mineral interaction properties were obtained, including the interaction energy and adhesion work of the water–mineral interface, mineral wettability, and the structural and diffusion properties of water molecules near the surface. Their simulation results revealed that the diffusion properties of water molecules on mineral surfaces played an important role in the wetting process.

Most studies focused on rocks with different chemical compositions. However, surface characteristics (such as cleavage plane, particle size and porosity), pH, and ionic strength were shown to determine the adsorption and self-assembly of complex molecules. Zhu et al. [23] studied the effect of rock surface roughness on the oil detachment process from a quartz surface in the presence of β-cyclodextrins (βCD). Fateme, S. et al. [6], obtained a silica surface model database for the full range of variable surface chemistry and pH (Q^2^, Q^3^, Q^4^ environments with adjustable degree of ionization), and explained the mechanistic details of the molecular adsorption of water vapor.

The number and arrangement of hydroxyl groups exposed on the mineral surface may also have an effect on the adsorption and detachment of oil molecules. Silica exists in different crystalline polymorphs and amorphous phases. The surface properties of different types of silica were related to the cleavage crystal plane, syntheses methods, roughness and thermal pretreatment. In this work, MD simulations were used to study the dynamic process of the aggregation and detachment of n-dodecane from silica surfaces with variable surface chemistry. Silica surfaces were obtained by mixing different crystalline silica surfaces (Q^2^, Q^3^, Q^4^ environments) and non-ionizing. The adsorption process and configurations of C_12_H_26_ molecules were simulated on different silica surfaces. Then, the detachment processes of C_12_H_26_ molecules from different silica surfaces were studied in the presence of sodium dodecyl sulphate (SDS) solutions.

## 2. Results

### 2.1. Adsorption of C_12_H_26_ on Different Silica Surfaces

**Figure 1:**
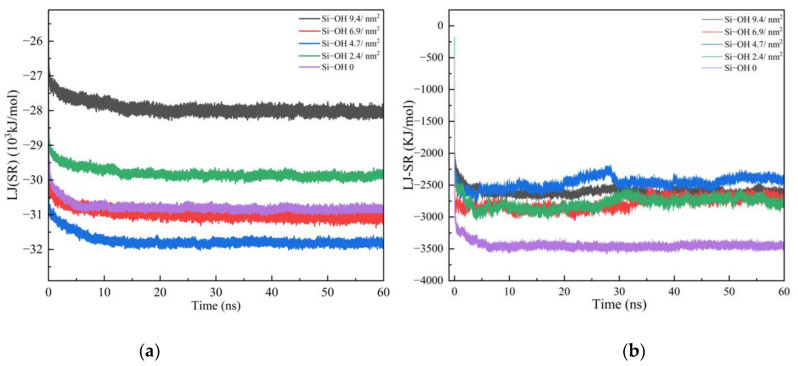
(**a**) The LJ (SR) energy profiles of C_12_H_26_ adsorption at different silica surfaces as a function of time. (**b**) The interaction energy between C_12_H_26_ and silica surfaces as a function of time during the adsorption process.

The arrangement of oil molecules on the solid surface is important for the oil detachment from the solid surface. At the beginning of the simulations, ninety C_12_H_26_ molecules were put above the silica surfaces. Figure 1a illustrated the variation of energy LJ (SR) between silica surfaces and oil molecules during the adsorption process of C_12_H_26_. The energy of five systems decreased at the beginning of the simulations. After 10 ns, the LJ energy profile of the systems reached a plateau, indicating that the five systems all reached equilibrium. Figure 1b compared the interaction energy between C_12_H_26_ and silica surfaces as a function of time during the adsorption process. The interaction energy between C_12_H_26_ and Si-OH 0 was lowest. The oil detachment from Si-OH 0 might be the most difficult, which can be proved by the final configurations of C_12_H_26_ detachment simulations at 50 ns and the oil and water density profiles.

The morphology of the oil adsorbed is related to the types and topography of the solid surfaces. In previous works [13], the well-ordered and layered oil molecules formed a close-packed structure on hydrophilic surfaces. Zhu et al. [23] reported that the interaction energy between oil and silica surfaces increased with the depth of the grooves.

**Figure 2:**
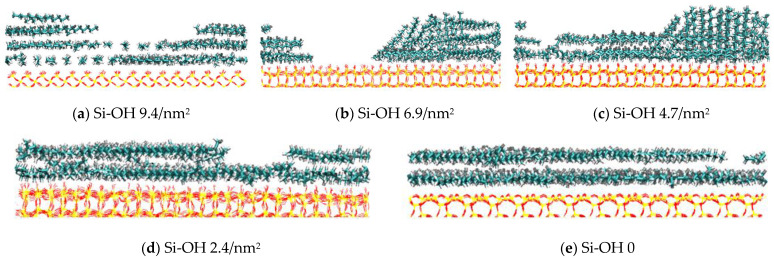
The side views of oil layers on different silica surfaces. Colors for atoms scheme are C_12_H_26_ (green), H (white), O (red), and Si (yellow).

**Figure 3:**
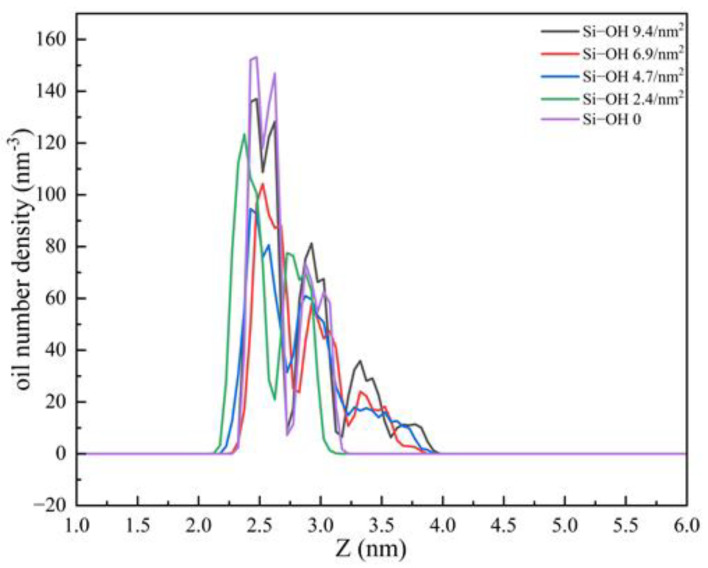
The number density profiles of C_12_H_26_ in the direction (z) normal to the different silica surfaces at 60 ns.

Side views of final configurations at different silica surfaces are shown in Figure 2. Five silica surfaces were used, with variable surface chemistry (Q^2^, Q^3^, Q^4^ environments), and which were cleaved on different crystal planes and had different types and area densities of silanol groups. For all the systems, C_12_H_26_ molecules were adsorbed on the SiO_2_ surfaces as layered structures. The first layer of C_12_H_26_ aligned parallel to the surface. There were four layers on the Si-OH 9.4/nm^2^. Figure 3 shows the number density profiles of C_12_H_26_ in the direction (z) normal to the different silica surfaces at 60 ns. The density profile of Si-OH 9.4/nm^2^ had four peaks. For Si-OH 6.9/nm^2^, the Si-OH 4.7/nm^2^ systems which were mixed crystalline, the density profiles had three peaks. The arrangement of C_12_H_26_ molecules can be divided into three layers (as shown in Figure 2b,c). The distribution of C_12_H_26_ molecules becomes more and more disordered on the second and third layers. For the Si-OH 2.4/nm^2^ and Si-OH 0 systems, there were two broad peaks on the density profiles, and the adsorptions of oil molecules can be divided into two layers (Figure 2).

**Figure 4:**
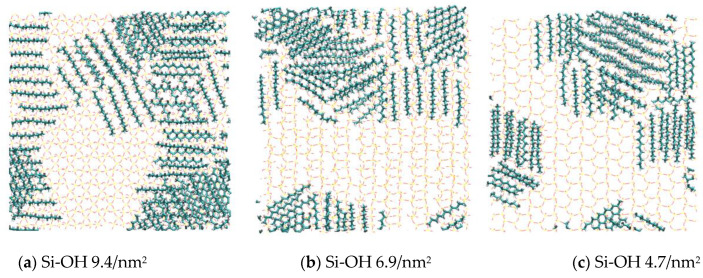

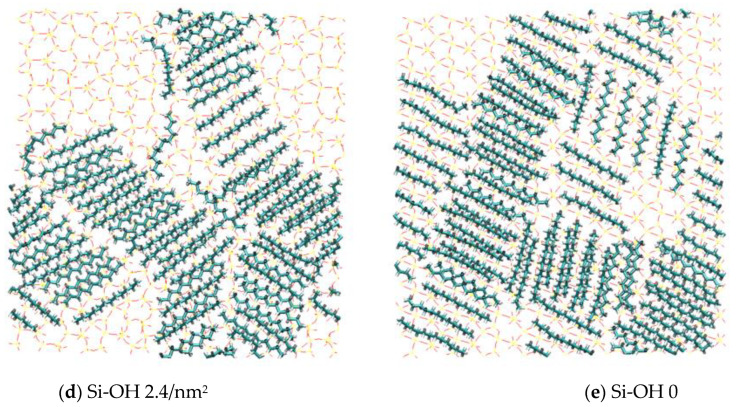
Final adsorption configurations of C_12_H_26_ molecules on different silica surfaces (top view). Colors for atoms scheme are C_12_H_26_ (green), H (white), O (red), and Si (yellow).

Top views of the final configurations of C_12_H_26_ after adsorption equilibrium are presented in Figure 4. Unlike the previous work [13], the first layer of oil molecules fully covered the silica surfaces. In this work, part of the silica surfaces was covered by C_12_H_26_ molecules. The rest of the oil molecules preferred to adsorb onto the first oil layer, rather than directly onto the silica surfaces. The distances between the first layers and surfaces were about 0.60 nm.

### 2.2. Detachment of C_12_H_26_ on Different Silica Surfaces

**Figure 5:**
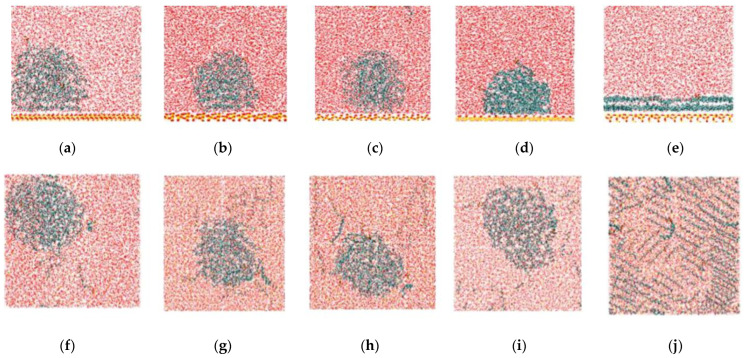
The final configurations of C_12_H_26_ detachment simulations at 50 ns (top view and side view). Colors for atoms scheme are C_12_H_26_ (green), H (white), O (red), and Si (yellow). Side views: (**a**) Si-OH 9.4/nm^2^ (**b**) Si-OH 6.9/nm^2^ (**c**) Si-OH 4.7/nm^2^ (**d**) Si-OH 2.4/nm^2^ (**e**) Si-OH 0. Top views: (**f**) Si-OH 9.4/nm^2^ (**g**) Si-OH 6.9/nm^2^ (**h**) Si-OH 4.7/nm^2^ (**i**) Si-OH 2.4/nm^2^ (**j**) Si-OH 0.

After the C_12_H_26_ molecules adsorbed on the silica surfaces, SDS solutions were added above the oil layers. After 50 ns simulations, the final configurations of the five systems are shown in Figure 5. For Si-OH 9.4/nm^2^, Si-OH 6.9/nm^2^, Si-OH 4.7/nm^2^ and Si-OH 2.4/nm^2^, the oil molecules can be partly detached. The oil layer cannot be detached from the Si-OH 0 surface.

Kolev et al. [24] carried out experiments on the detachment of oil drops from glass substrates, and obtained results showing that the three-phase (solid–oil–water) contact line shrank spontaneously in anionic surfactant solutions, and eventually the oil drop detached from the substrate. The shrinking of the three-phase s contact line was due to the molecular penetration (diffusion) of the water molecules between the oil drop and the solid phase. The hydrogen bonds between the solid surface and water molecules are vital for the formation of water channels and the diffusion of water onto the solid surface. The Si-OH 0 system is a Q^4^ crystalline type which is prepared from the Q^3^ surface by complete condensation of surface silanol groups at high temperature. No silanol groups were on the Si-OH 0 surface. Few H-bonds can form on the Si-OH 0 surface, so water molecules cannot penetrate through the oil layer and cannot diffuse at the Si-OH 0 surface. The oil arrangement of the first layer on the Si-OH 0 surface did not change after the addition of the surfactant solution. Oil molecules cannot be replaced by water molecules on the Si-OH 0 surface (as shown in Figure 5).

**Figure 6:**
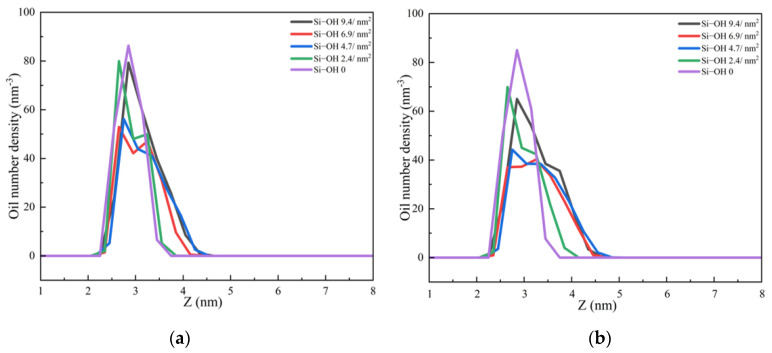

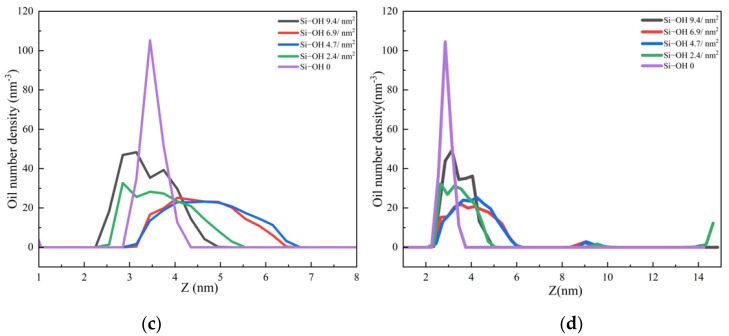
The number density profiles of the C_12_H_26_ molecules in the direction (z) perpendicular to the surface at different times. (**a**) 500 ps (**b**) 1 ns (**c**) 5 ns (**d**) 10 ns.

The number density profiles of the C_12_H_26_ molecules in the direction (z) perpendicular to the surface at different times are shown in Figure 6. For Si-OH 6.9/nm^2^ and Si-OH 4.7/nm^2^, the first peak of the oil number density curves decreased and disappeared at 5 ns. For Si-OH 9.4/nm^2^ and Si-OH 2.4/nm^2^, the first peaks of the oil number density curves decreased much slower than for the Si-OH 6.9/nm^2^, Si-OH 4.7/nm^2^. For Si-OH 0, the peak of oil number density at 3.15 nm did not decrease after 50 ns detachment simulations. These results agreed well with the final configurations of oil molecules at the end of the simulations. At the Si-OH 0, the arrangement of the first oil layer did not change.

**Figure 7:**
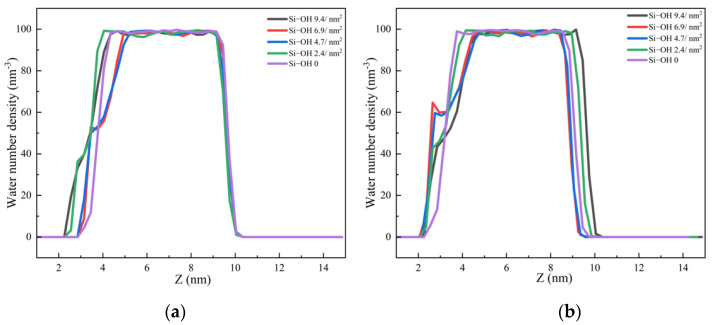

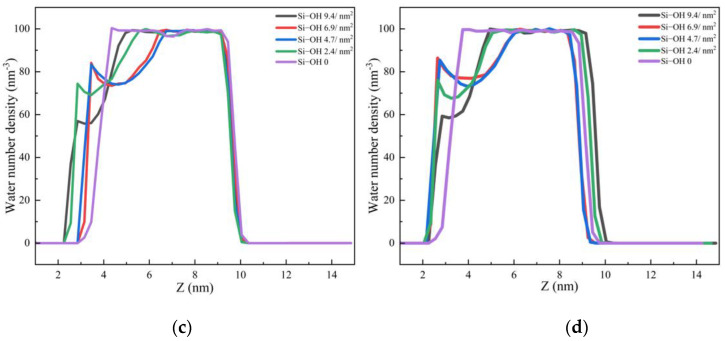
The number density profiles of the water molecules in the direction (z) perpendicular to the surfaces at different times. (**a**) 500 ps (**b**) 1 ns (**c**) 5 ns (**d**) 10 ns.

The number density profiles of water molecules in the direction (z) perpendicular to the surface at different times are shown in Figure 7. At the beginning of the simulations, the number densities of the water molecules were zero below the oil layer (z < 2.5 nm). The key to oil detachment on a hydrophilic surface is the formation of water channels and the diffusion of water on the three-phase contact line [13]. Once the water channels are formed, the oil will be detached rapidly, due to the weak interaction between the alkane molecules and silica surfaces. At 500 ps, new peaks emerged at silica surfaces for the Si-OH 6.9/nm^2^, Si-OH 4.7/nm^2^ systems. At 1ns, new peaks emerged for the Si-OH 9.4/nm^2^ and Si-OH 2.4/nm^2^. H-bonds are the main driving force for the formation of water channels and the diffusion of water molecules on the silica surfaces. Under the H-bonds between the water molecule and the silica surface, water penetrated through the oil layer and the water channel formed through the oil layers. The density of water molecules at the silica surface increased with the evolution of time.

Once the water-channel was formed, water molecules diffused on the solid surfaces which contained Q^2^ and Q^3^ surface environments. The solid–oil–water contact line was shrinking. The oil molecules were replaced by water molecules. The number densities of oil decreased as a function of time, as shown in Figure 6. The water channel became wider. More water molecules entered into the solid surface, and the new peaks of the water density profiles increased as a function of time (Figure 7c,d). Unlike the previous work [13], water number densities at the solid surfaces were lower than those in the bulk. The continuous water film could not form at the end of the simulations, and the oil did not completely detach. In contrast, a new peak of the water number density profile did not emerge at the Si-OH 0 surface. The water could not diffuse and could not replace the oil molecules on the Si-OH 0 surface.

**Figure 8:**
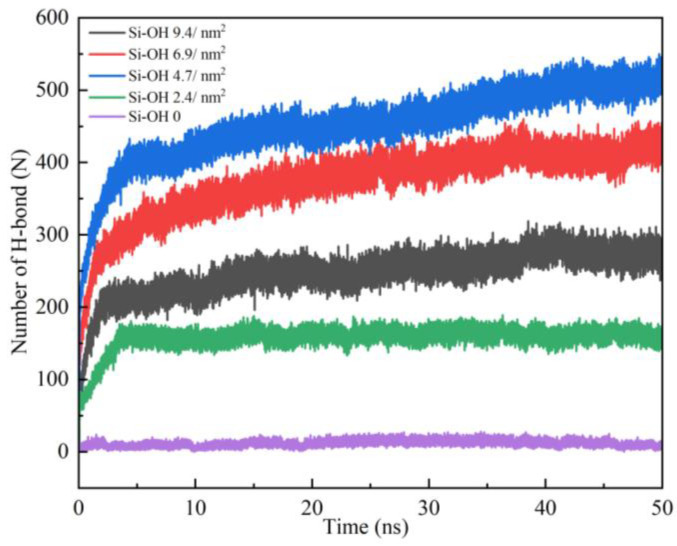
The number of hydrogen bonds formed between water molecules and SiO_2_ surface as a function of time.

**Figure 9:**
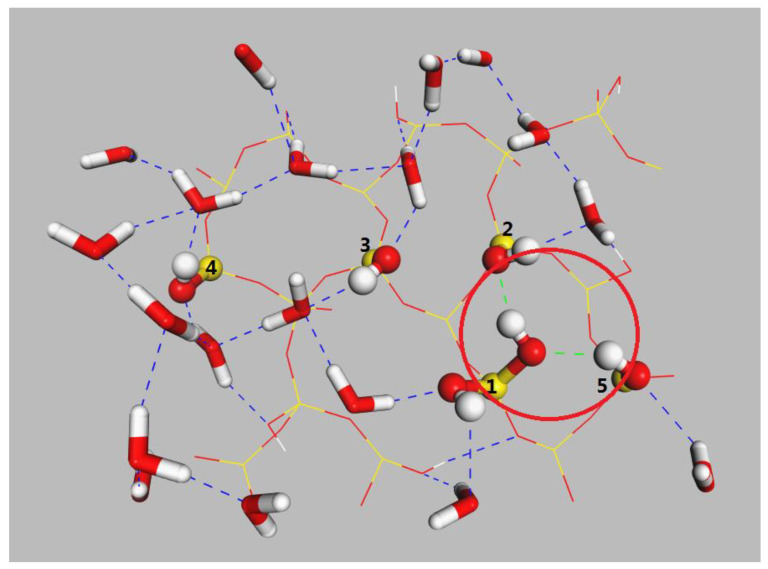
H-bonds formed on Si-OH 6.9/nm^2^ surface at 50 ns. Colors for atoms scheme are H (white), O (red), and Si (yellow), H-bonds between water and silanol groups (blue), H-bonds among silanol groups (green). Si(1) is a Q^2^ type. Si(2), Si(3), Si(4), and Si(5) are Q^3^ types.

**Figure 10:**
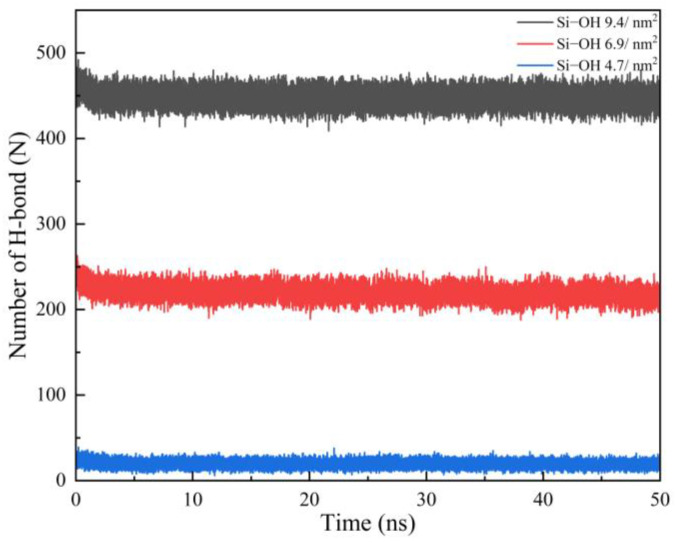
The number of hydrogen bonds among silanol groups as function of time.

Figure 8 shows the H-bond number profiles between the water molecules and silica surfaces as a function of time during the oil detachment simulations. Although Si-OH 9.4/nm^2^ and Si-OH 6.9/nm^2^ had more silanol groups pre nm^2^, they all contained Q^2^ surface environments, which had two silanol groups per superficial silicon atom (=Si(OH)_2_). Figure 9 shows the H-bonds on the Si-OH 6.9/nm^2^ surface at 50ns. Si-OH 6.9/nm^2^ (Figure 10) contained mixed Q^2^/Q^3^(1:1) surface environments. As shown in Figure 9, Si(1) is a Q^2^ type. Si(2), Si(3), Si(4), and Si(5) are Q^3^ types. Silanol groups on Si(1) formed H-bonds with both water and silanol groups of Si(2), Si(5). However, the silanol groups of Si(3) and Si(4) only formed H-bonds with water. When the area density of the silanol groups increased from 4.7 to 9.4 per nm^2^, the surface contained 50–100% Q^2^ surface environments. The number of hydrogen bonds among the silanol groups increased when the surface contained more Q^2^ surface (Figure 10), which hindered the diffusion of water. H-bonds formed among silanol groups at Si-OH 9.4/nm^2^ were more than Si-OH 6.9/nm^2^. The shrinking of the oil–water–solid contact line became slower as the silanol numbers and Q^2^ contents increased.

Si-OH 4.7/nm^2^ are perfect Q^3^ crystalline, which has one silanol group per superficial silicon atom (≡Si(OH)) type and the distribution of silanol groups are well-ordered (as shown in Figure 10). The H-bond number between Si-OH 4.7/nm^2^ and water increased more quickly than that on Si-OH 9.4/nm^2^ and Si-OH 6.9/nm^2^. Oil molecules detached faster on Si-OH 4.7/nm^2^. For Si-OH 2.4/nm^2^, which contained Q^3^/Q^4^ mixed surface environments, the H-bond number among the silanol groups was zero. Si-OH 2.4/nm^2^ had fewer silanol groups, the H-bond number between the water and silanol groups increased slowly, and the H-bond number was less than for Si-OH 9.4/nm^2^, Si-OH 6.9/nm^2^. Water molecules diffused faster on Si-OH 9.4/nm^2^ and Si-OH 6.9/nm^2^ than on Si-OH 2.4/nm^2^. However, the Si-OH 0 surface had no silanol groups on the surface, and few H-bonds formed. Water diffusion was difficult on the Si-OH 0 surface, so the solid–oil–water contact line did not shrink. The water could not replace the oil molecules on the Si-OH 0 surface.

## 3. Discussion

The shrinking of the oil–water–solid contact line was a key step in the oil detachment, due to water diffusion on the three-phase contact line. The formation of H-bonds was one of the driving forces for water diffusion on the solid surface. Silica exists in various crystalline polymorphs and amorphous phases. The type and area density of silanol groups depends on the crystal plane, particle size, synthesis protocol, thermal pretreatment, roughness and porosity.

**Figure 11:**
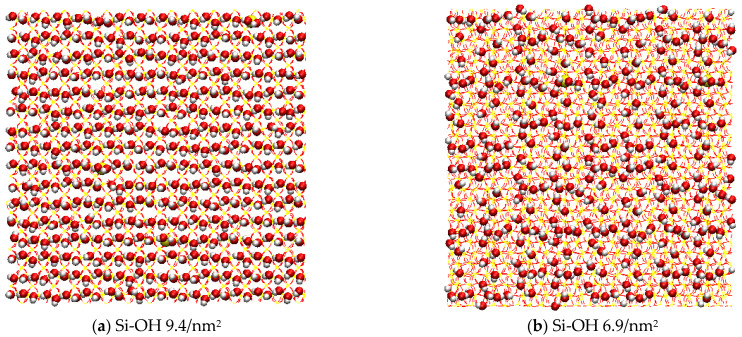

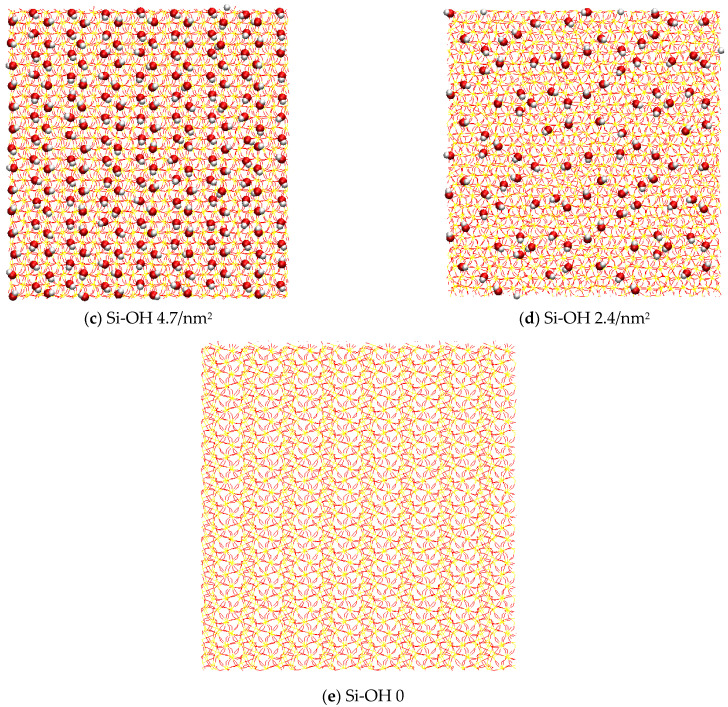
Top views of different silica surfaces. Colors for atoms scheme are H (white), O (red), and Si (yellow).

In this work, silica surfaces were used with average area density of silanol groups of between 9.4 and 0 per nm^2^ and contained variable Q^2^, Q^3^, Q^4^ surface environments. Some hydrated cleavage planes of quartz, surfaces of large silica nanoparticles, and various forms of silica at high pH contain Q^2^ surface environments (Figure 11a) and mixed Q^2^/Q^3^ surface environments (Figure 11b). The area density of the silanol groups is in the range of 9.4 to 4.7 per nm^2^. The silanol groups of the Q^2^ surface were (-Si(OH)_2_), which was two silanol groups per superficial silicon atom. H-bonds could be formed among the silanol groups on the Q^2^ surface. When the area density of the silanol groups increased from 4.7 to 9.4 per nm^2^, the surface contained 50-100% Q^2^ surface environments. The shrinking of oil–water–solid contact line became slower as the silanol numbers and Q^2^ contents increased.

A perfect Q^3^ surface contains one silanol group per superficial silicon atom (=Si(OH) and 4.7 Si-OH groups per nm^2^ (Figure 11c). Few H-bonds could be formed among silanol groups on the Q^3^ surface (as shown in Figure 10). H-bonds preferred to form between water and silanol groups (as shown in Figure 8), which promoted the diffusion of water on the Q^3^ surface. The oil detachment was easier and faster on Si-OH 6.9/nm^2^ and 4.7/nm^2^ than on Si-OH 9.4/nm^2^.

A pure Q^4^ surface model (Figure 11e) was prepared from a Q^3^ surface by complete condensation of the surface silanol groups at high temperature. The silanol density of the Q^4^ surface was zero. Few H-bonds could be formed between the water and silanol groups on the Q^4^ surface. Water cannot diffuse and oil cannot detach on the Q^4^ surface.

Silica surfaces and nanoparticles annealed at 200–1000 °C comprise Q^2^ and Q^4^ environments, and the average area density of the surface silanol groups is in the range of 4.7 to 0 per nm^2^. Si-OH 2.4/nm^2^ were mixed Q^3^/Q^4^ environments (Figure 11d). H-bond numbers between the water and silanol groups of Si-OH 2.4/nm^2^ were much less than for the Q^2^/Q^3^ surface, and so the three-phase contact line of Si-OH 2.4/nm^2^ shrank slowly, and less oil could be removed.

## 4. Materials and Methods

### 4.1. Model Systems

In this work, the C_12_H_26_ was used as an ideal model of the oil molecule. The C_12_H_26_ molecule and dodecyl sulfate ion were constructed using Avogadro-1.2.0n software. Silica surfaces were from Fateme’s work [6] and neutrally charged. The Q^2^ surface was derived from the (100) cleavage plane of α-quartz, and contained 9.4 Si-OH groups per nm^2^. The Q^3^ surface models were derived from the (101) cleavage plane of α-cristobalit, and contained 4.7 Si-OH groups per nm^2^. The pure Q^4^ surface model was prepared from the Q^3^ surface by complete condensation of the surface silanol groups at high temperature. After energy minimization, some Si-O bonds stretched 10% and the density of the silanol groups was zero.

The SiO_2_ surfaces are shown in Figure 11. Figure 11a–e indicated the hydroxylation densities of silicon surfaces of 9.4/nm^2^, 6.9/nm^2^, 4.7/nm^2^, 2.4/nm^2^ and 0, respectively. Si-OH 9.4/nm^2^ are Q^2^ crystalline, and contained 9.4 Si-OH groups per nm^2^. Si-OH 6.9/nm^2^ are Q^2^ and Q^3^ mixed crystalline, and contained 6.9 Si-OH groups per nm^2^. Si-OH 4.7/nm^2^ are Q^3^ crystalline, and contained 6.9 Si-OH groups per nm^2^. Si-OH 2.4/nm^2^ was the Q^3^ and Q^4^ mixed crystalline type, and the Si-OH group density was 2.4 per nm^2^. Si-OH 0 was the Q^4^ crystalline type, and there were no Si-OH groups on the surface. The silica surface model box size was about 6.9 × 6.9 × 10.0 nm^3^. The energy minimization and equilibrium simulations were performed on five surfaces before the simulation process, to ensure the stability of the surface structure.

### 4.2. Computational Details

GROMMACS 2019.3 software package was employed to carry out all the MD simulations. The all-atom optimized performance for the liquid systems (OPLS-AA) force field [25,26,27] was adopted for all the potential. The simulation parameters for SDS and oil molecules used in this study were derived from the AMBER force field [28]. Water molecules were described by the simple point charge/extend (SPC/E) model [29]. The atomic types and atomic charges of SiO_2_ in this paper are given in Table 1.

At the beginning of the simulations, ninety C_12_H_26_ molecules were put above each silica surface. After the adsorption of C_12_H_26_ molecules, the last frame of the trajectory was used in the detachment process. SDS solutions were added into the systems. Each of the systems was firstly minimized, using the steepest descent method. After the minimization, MD simulations (NVT) were carried out under canonical ensemble. Periodic boundary conditions were applied in the x, y, and z directions. For each system, the absorption and detachment process simulations were performed for 60 ns and 50 ns with a time step of 2 fs. The temperature was set to 300 K. The VMD 1.9.3 package was used for visualization More details of the parameters for the simulation systems are given in Table 2.

## 5. Conclusions

The area density and types of silanol groups played a role in the properties of the silica surfaces. In this paper, the adsorption and detachment processes of dodecane molecules were simulated on five silica surfaces with Q^2^, Q^3^, and Q^4^ surface environments. The results demonstrated that more C_12_H_26_ were adsorbed on the Q^4^ crystalline type. When the surfaces contained more Q^2^ crystalline type which had (≡Si(OH)_2_)-type silanol groups, less oil detached, due to the formations of H-bonds among the silanol groups. More oil detached on the Si-OH 4.7/nm^2^, which were Q^3^ crystalline type and had (≡Si(OH))-type silanol groups. There were no silanol groups on the Si-OH 0 surface, even though, in the presence of the surfactant solutions, the water cannot diffuse and the oil cannot detach from the Si-OH 0 surface. The area density and type of silanol groups both affect the formation of water channels and the diffusion of water on the silica surfaces, which are essential and fundamental steps for the liberation of oil from reservoir solids or host rocks.

## Figures and Tables

**Table 1:** Atom type symbols and atomic charges of silica used in this work.

Atom	Atom Type	Charge (e)
Si	SC4	1.1
Si-O-Si	OC23	−0.55
Si-O-H	OC24	−0.675
H	HOY	0.4

**Table 2:** Details of simulation systems.

	C_12_H_26_/N	SOL/N	SDS/N
Si-OH 9.4/nm^2^	90	9964	5
Si-OH 6.9/nm^2^	90	8941	5
Si-OH 4.7/nm^2^	90	8890	5
Si-OH 2.4/nm^2^	90	9650	5
Si-OH 0	90	9096	5

## Data Availability

The data that support the findings of this study are available from the corresponding author upon reasonable request.
